# Sleep-independent offline consolidation of response inhibition during the daytime post-training period

**DOI:** 10.1038/srep10362

**Published:** 2015-05-20

**Authors:** Motoyasu Honma, Takuya Yoshiike, Hiroki Ikeda, Kenichi Kuriyama

**Affiliations:** 1 ^1^Department of Adult Mental Health, National Institute of Mental Health, National Center of Neurology and Psychiatry, 4-1-1 Ogawa-Higashi, Kodaira, Tokyo 187-8502, Japan; 2 ^2^Department of Neurology, Showa University School of Medicine, 1-5-8 Hatanodai, Shinagawa-ku, Tokyo 142-8666, Japan; 3 ^3^Laboratory of Neuromodulation, Kanagawa Psychiatric Center, 2-5-1 Serigaya, Kohnan-ku, Yokohama, Kanagawa 233-0006, Japan; 4 ^4^Department of Psychiatry, Shiga University of Medical Science, Seta Tsukinowa-cho, Otsu, Shiga 520-2192, Japan

## Abstract

Appropriate inhibitory response control is associated with goal-directed behavior. Sleep accelerates the offline consolidation of acquired motor skills that are explicitly predictable; however, the effect of sleep on implicit (unpredictable) motor skills remains controversial. We speculated that a key component of response inhibition skill differentiates between these skill consolidation properties because explicit prediction can minimize the inhibitory efforts in a motor skill. We explored the offline skill learning properties of response inhibition during sleep and wakefulness using auditory Go and Go/Nogo tasks. We attempted to discriminate the possible effects of time elapsed after training (12 or 24 h), post-training sleep/wake state (sleep or wakefulness), and time of day (nighttime or daytime) in 79 healthy human subjects divided into 6 groups that underwent various sleep regimens prior to training and retesting. We found that delayed response inhibition skill improvement was achieved via a simple passage of daytime, regardless of the participants’ alertness level. Our results suggest that sleep-independent neuroplasticity occurs during the daytime and facilitates a delayed learning of response inhibition skill.

Appropriate inhibitory response control leads to improved goal-directed behavior and reflects an important aspect of psychosocial adaptation to daily life^1^,^2^,^3^. Superior inhibitory response control skills enable us to alter a planned action to an appropriate one in response to an unpredictable change in circumstances. Response inhibition (RI), the ability to inhibit preplanned or ongoing motor action, requires both executive function and subsidiary sensorimotor functions for task switching, control impulsiveness, prepotent response suppression, category shifting, and prioritizing actions toward an appropriate goal^4^,^5^. RI immaturity or impairment is considered to be involved in the pathology of various mental disorders, such as attention-deficit/hyperactivity disorder^6^,^7^ and addictions to gaming, gambling, and alcohol^8^. Because RI skill improvements could result in better psychosocial adaptation^6^,^7^,^8^ and augment psychiatric treatment, developing efficient RI training is an urgent priority.

Stop-signal tasks^9^,^10^, including the Go/Nogo task^4^,^11^,^12^,^13^, are widely used to measure RI in experimental settings. This type of task comprises a major response-accelerating stimulus (e.g., Go or imperative) and a minor response-inhibiting stimulus (e.g., Nogo or stop signal). Although the responses to Go stimuli chiefly reflect a motor acceleration controlled by the motor cortex, the invisible responses to Nogo stimuli, which are closely associated with cognitive inhibition elicited by the central executive system in the prefrontal cortex (PFC)^14^,^15^, affect the response to the Go stimuli. Thus, Go/Nogo task performance is able to reflect cognitive inhibition ability better than performance on a simple response task.

Sleep has been shown to have a beneficial role in humans’ offline learning of motor procedural skills^16^,^17^,^18^,^19^. Walker *et al.* clearly demonstrated that a night of sleep after skill training results in significant improvements of a motor sequence tapping (MST) skill on a finger-tapping task^17^, whereas equivalent periods of standby time during wakefulness did not affect participants’ MST skills. Furthermore, a distinctive sleep-dependent MST skill learning profile emerged from the initial practice-dependent improvement during training^18^,^20^, suggesting a separate ongoing process of offline MST skill consolidation from the initial acquisition process. In addition to a full night’s sleep, short daytime naps have been suggested to actively contribute to offline MST skill consolidation^21^,^22^,^23^,^24^. Mednick *et al.* reported that a brief (60–90 min) daytime nap facilitated an equivalent offline improvement on MST skill^24^ that was comparable to that following an entire night of sleep^16^,^17^, regardless of the time of day of the sleep period.

However, the role of sleep in the offline consolidation of motor procedural skills is still debated. Song, Howard, and Howard reported that a probabilistic motor (PM) skill examined with a serial response time task did not improve following sleep in offline PM skill consolidation^25^. Rather, the PM skill was enhanced to a greater extent after a daytime waking time period had elapsed.

Here, we explore the offline skill consolidation property of RI. A previous study showed that approximately 30-min training immediately improved RI skill, but it did not lead to benefits 4 months after the training^26^. However, this does not eliminate the possibility of a delayed overnight (offline) improvement in RI skill because the improvement would deteriorate during the 4-month interval without any continuous practice^20^. It has been shown that working memory (WM) and RI partially share neurocognitive resources such as the left inferior parietal lobule (IPL), right dorsolateral prefrontal cortex (DLPFC), inferior frontal gyrus (IFG), left posterior lateral frontal cortex, and supplementary motor area (SMA) to performing flexible and adaptive behavior^27^,^28^, and WM skills are consolidated offline^29^. The offline consolidation of WM skills is associated with increased activity in the middle frontal gyrus and superior and inferior parietal cortices after 5 weeks of WM training^30^.

To understand the contribution of offline consolidation of RI, we simultaneously considered the effect of sleep state, the crude effects of time of day (nighttime or daytime) of the elapsed time period between training and retest, and an interaction between sleep and time of day during offline learning of the RI skill across a day. We utilized an auditory Go/Nogo task that simultaneously measured response time (RT) and hit rate (HR) as indexes of prepotent RI abilities (speed and accuracy) as compared with those in the Go (simple response task) task. The mnemonic property of RI could contribute to psychosocial rehabilitation for various psychiatric disorders.

## Results

### Sleep habits and experimental sleep control

**Figure 1:**
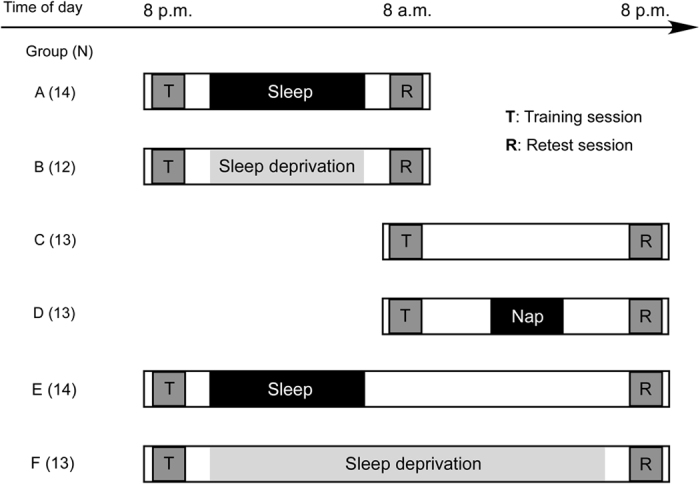
Experimental schedule. **** Time course of the experimental schedule for each group. The intervals between training and retest sessions were 12 h in groups **A**–**D** and 24 h in groups **E** and **F**. Groups **A**, **B**, **E**, and **F** were trained in the evening (8 p.m.), and groups **C** and **D** were trained in the morning (8 a.m.). Groups **A** and **E** slept at night as usual, but groups B and F were sleep deprived. Groups **C** and **D** performed the experiment in the daytime, whereas group **D** took a 3-h nap during the training-retest interval (from 1–4 p.m.).

We attempted to discriminate the possible effects of time elapsed after training (12 or 24 h), the post-training sleep/wake state (sleep or wakefulness), and time of day (nighttime or daytime) in 79 healthy human subjects divided into 6 groups that performed various sleep regimens prior to training and retesting (Fig. 1). One-way analyses of variance (ANOVAs) did not show any significant group differences in habitual sleep parameters of sleep onset (F_(5, 78)_ = 0.428, *p* = 0.733), waking hours (F_(5, 78)_ = 0.524, *p* = 0.758), or sleep duration (F_(5, 78)_ = 0.624, *p* = 0.682; Table 1). Participants in groups A, D, and E slept during the intersession interval for an average of 7.06 ± 0.21 h (range, 5.11–7.81), 2.49 ± 0.07 h (range, 2.16–2.96), and 7.05 ± 0.22 h (range, 5.12–7.80), respectively.

### Sleepiness level

**Figure 2:**
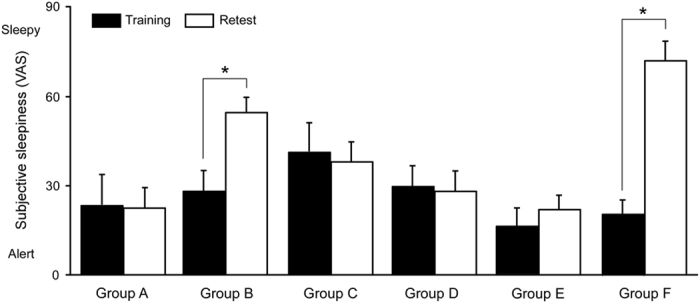
Changes in subjective sleepiness across training-retest intervals. **** The subjective sleepiness ratings in groups B and F measured by the visual analog scale (VAS) at the retest session were significantly higher than those at the training session, presumably due to total sleep deprivation during the post-training night. The bars and error bars represent the means and SEMs, respectively. *p < 0.05.

A two-way ANOVA revealed a significant main effect of group (F_(5,73)_ = 5.58, *p* < 0.001) and a group×session interaction (F_(5,73)_ = 7.27, *p* < 0.001) but showed a non-significant main effect of session (F_(5,73)_ = 0.97, *p* = 0.489). Subsequent multiple comparisons revealed that participants in groups B (*p* < 0.001) and F (*p* < 0.001) felt significantly greater sleepiness during the retest session compared to the training session (Fig. 2), which might have been due to sleep deprivation (SD). However, subjective sleepiness level did not seem to immediately affect Go and Go/Nogo task performance. We did not observe significant correlations between subjective sleepiness and RT in Go (*r* = −0.199, *p* = 0.078) and Go/Nogo (*r* = −0.161, *p* = 0.154) trainings or Go (*r* = 0.088, *p* = 0.436) and Go/Nogo (*r* = 0.196, *p* = 0.081) retests. Similarly, we did not note a significant correlation between subjective sleepiness and HR in Go (*r* = 0.153, *p* = 0.176) and Go/Nogo (*r* = −0.179, *p* = 0.112) trainings or Go (*r* = 0.088, *p* = 0.436) and Go/Nogo (*r* = −149, *p* = 0.186) retests.

### Immediate learning of RI

Lower performances in the Go/Nogo task compared to the Go task suggested appropriate cognitive load by RI on Go/Nogo task performance. The RT on the Go/Nogo training (mean, 379 ms; SEM, 4.09 ms) was significantly slower (*t*_(78)_ = 11.917, *p* < 0.0001) than that on the Go training (mean, 316 ms; SEM: 5.51 ms). The HR on the Go/Nogo training (mean, 97.28%; SEM, 0.56%) was significantly lower (*t*_(78)_ = 4.499, *p* < 0.0001) than that on the Go training (mean, 99.06%; SEM, 0.29%).

**Figure 3:**
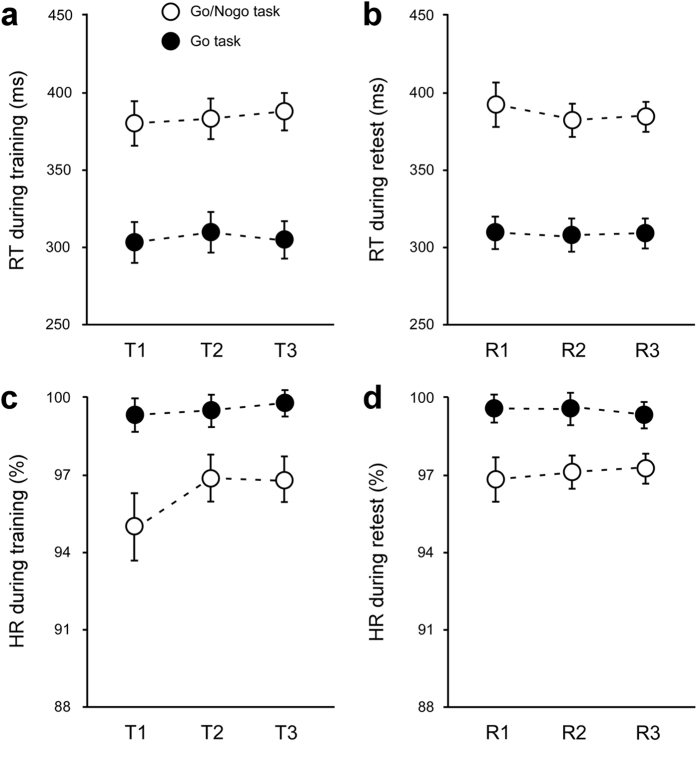
Immediate training profiles on response time (RT) and hit rate (HR) at training and retest sessions. **** There were no significant differences among the three blocks of Go and Go/Nogo tasks during the training (**a**) or retest sessions (**b**) with regard to RTs or the training (**c**) and retest sessions (**d**) with regard to HRs. Open circles and error bars represent the means and SEMs on Go/Nogo performances, respectively, and filled circles and error bars represent means and SEMs on Go performances, respectively.

No immediate within-training effects for RT and HR on the Go/Nogo and Go tasks were observed during the training and retest sessions in any of the groups. Two-way ANOVAs showed non-significant main effects of group and training block and non-significant group × training-block interactions for both RT and HR on the Go/Nogo (RT: *p* > 0.25, HR: *p* > 0.20) and Go (RT: *p* > 0.25, HR: *p* > 0.20) tasks at the training and retest sessions (Fig. 3a–d). In addition, unpaired *t*-tests showed non-significant differences in training RT (Go: *t*_(77)_ = 1.426, *p* = 0.158; Go/Nogo: *t*_(77)_ = 0.780, *p* = 0.438) and training HR (Go: *t*_(77)_ = 0.762, *p* = 0.449; Go/Nogo: *t*_(77)_ = −0.051, *p* = 0.960) caused by training time of day (8 a.m. vs. 8 p.m.). These results suggested that RI skill was not immediately developed by repetitive training and was not affected by the diurnal difference in task execution (8 a.m. or 8 p.m.).

### Delayed RI skill learning

**Figure 4:**
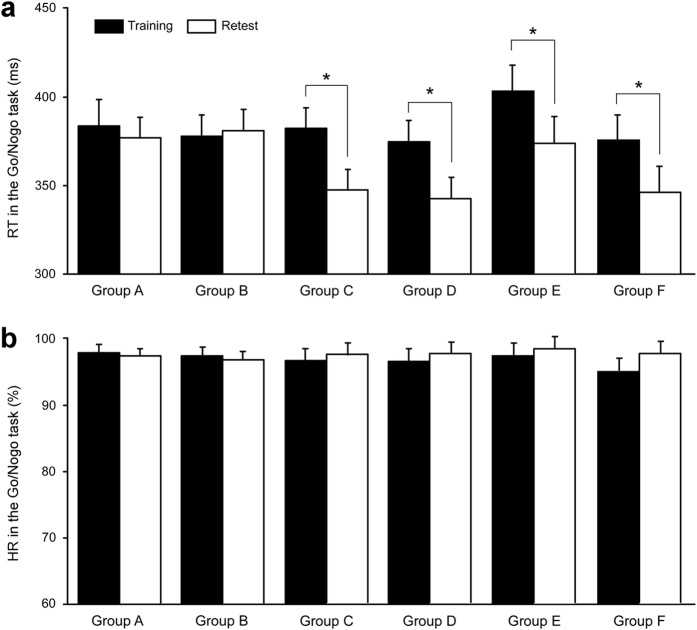
Delayed improvement for Go/Nogo performances. **** (**a**) Signific**a**nt delayed improvements in Go/Nogo response time (RT) were seen in groups C–F. (**b**) Delayed improvements in Go/Nogo hit rate (HR) were not observed in any group. Bars and error bars represent the means and SEMs, respectively. *p < 0.05.

A two-way ANOVA for Go/Nogo RT revealed a significant main effect of session (F_(1,73)_ = 14.29; *p* < 0.001) and a significant group × session interaction (F_(5,73)_ = 4.73; *p* < 0.001), but it showed a nonsignificant group effect (F_(5,73)_ = 1.842; *p* = 0.115). Post hoc tests revealed that the Go/Nogo RT in the retest session was significantly faster than that in the training session in groups C (*p* < 0.001), D (*p* < 0.001), E (*p* = 0.049), and F (*p* = 0.021; Fig. 4a). Go/Nogo HR reached approximately 100% regardless of the session and group. A two-way ANOVA for Go/Nogo HR showed nonsignificant effects of session and group and a nonsignificant group×session interaction (all *p* > 0.10; Fig. 4b).

No significant differences in the Go/Nogo RT improvement rate were observed among groups C–F. A one-way ANOVA showed no significant main effect of group on the Go/Nogo RT improvement rate (F_(3, 52)_ = 0.755, *p* = 0.525).

In contrast, Go task performances did not show any significant delayed improvements in any of the groups. A two-way ANOVA for Go RT showed nonsignificant main effects of session and group and a nonsignificant group×session interaction (all *p* > 0.10; see Table 2). The Go HR also reached approximately 100% regardless of session and group. A two-way ANOVA for Go HR showed nonsignificant main effects of session and group and a nonsignificant group×session interaction (all *p* > 0.10).

**Figure 5:**
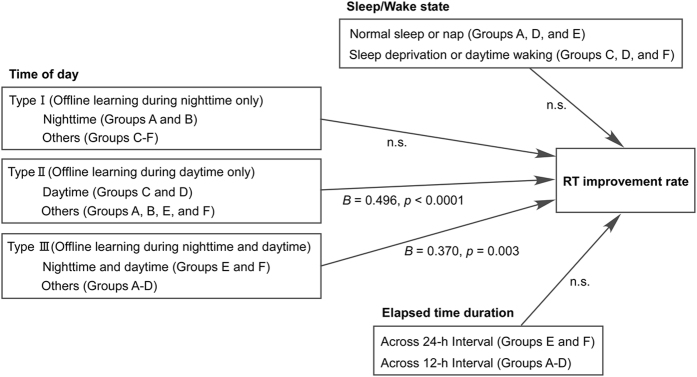
Intersession differences in % improvement in response time (RT) on the Go/Nogo task. **** A stepwise multiple linear regression analysis revealed that the daytime (Type II) and whole-day (Type III) denominators of time of day were predictive of the Go/Nogo RT improvement rate. None of the other variables of sleep/wake state, elapsed time duration, or the nighttime (Type I) denominator of time of day from predictive variables of the Go/Nogo RT improvement rate was significant. No interactions between predictive variables were observed.

A multiple regression analysis identified daytime (*B* = 0.496, *p* < 0.0001) and whole-day (*B* = 0.370, *p* = 0.003) denominators of time of day as predictive variables of the Go/Nogo RT improvement rate and did not show any significant differences in the contribution ratio between these denominators (*p* > 0.05; Fig. 5). However, it excluded all the other variables of sleep state (sleep or wakefulness), training–retest interval (12 or 24 h) and the nighttime denominator of time of day as predictive variables of the Go/Nogo RT improvement rate. In addition, no interactions between predictive variables were observed (all *p* > 0.10). A high determination coefficient of the model with low collinearity was achieved by these denominators (R^2^ = 0.196, Fig. 5).

## Discussion

Our results suggest that RI skill in an auditory Go/Nogo task improved during the daytime but not the nighttime offline period, and that this occurred regardless of the brain’s sleep/wake state. Here we discuss several confounding factors that could have influenced our results.

The response speed (RT) seemed to be a more sensitive indicator of prepotent RI ability than accuracy (HR), which is in line with the results of previous studies^4^,^11^,^12^,^13^. Response speed is also more sensitive to the delayed (offline) learning properties of various motor skills compared to accuracy^25^,^31^, including sleep-dependent skill consolidation^17^,^18^,^21^. Although immediate training did not result in Go and Go/Nogo performance improvements, a greater delayed improvement of Go/Nogo RT was observed not after an elapsed nighttime period but after an elapsed daytime period regardless of sleep/wake state, elapsed time duration, or alertness. The current results suggested that RI skill exhibited a peculiar diurnal variation in offline learning profiles without sleep/wake state dependency; this is different from other motor skills^17^,^25^,^32^, including those closely engaged in PFC function^29^. This conclusion was arrived at after ruling out plausible influences of time of day, elapsed time duration between training and retesting, and alertness (including possible acute stress of sleep deprivation effects on the Go and Go/Nogo task performances).

**Figure 6:**
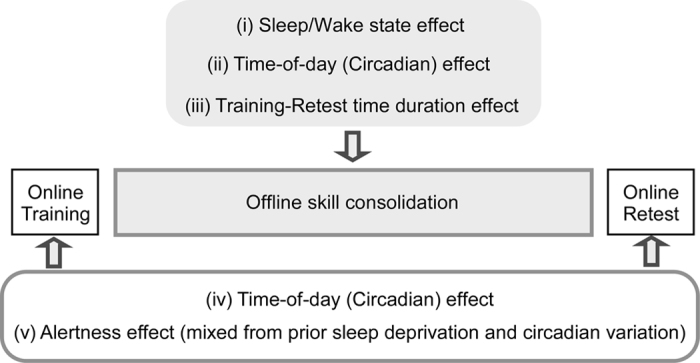
Possible confounding factors in immediate (online) task performances and delayed (offline) skill consolidation. **** The confounding factors (**i**–**v**) could manipulate training–retest Go/Nogo task performance. Although the statistical analyses results demonstrate that the offline improvement in Go/Nogo response time (RT) seems to be directly influenced by the time-of-day effect (**ii**), the potent time-of-day effect on the online task performance of Go/Nogo RT could make it appear as though there was offline improvement (**iv**). The effects of sleep/wake state (**i**), training–retest t**i**me duration (**iii**) on offline RI skill consolidation, and the effect of alertness (**v**) on online RI performances were statistically ruled out.

Skill learning has been considered to undergo two distinct processes: an immediate (online) within-session process of skill training and a delayed (offline) across-session process of skill learning without ongoing skill training^33^,^34^ (see Fig. 6). In the current study, trial repetition within a training session did not improve Go or Go/Nogo performance regardless of the time of day of the initial training session.

We did not find any significant time-of-day effects in subjective sleepiness or task performance, but we did observe significant effects of SD on these variables. SD clearly deteriorated subsequent sleepiness but not Go and Go/Nogo performance, although definite trends in sleepiness effects on task performance were observed. Retest Go performance in groups B and F was not deteriorated even though both groups showed greater subjective sleepiness. Previous studies suggested that alertness had a stronger effect on accuracy than on the speed of a simple response task^35^,^36^; accuracy was deteriorated by deprivation of a night’s sleep, but speed was not significantly altered^35^,^37^,^38^. However, previous studies used a simple response task that required longer trial repetitions (60 or more) of motor responses; thus, we could not detect a significant influence of SD on Go or Go/Nogo task performance. In other words, alertness has a minimal influence on task performances when a single trial block includes short trial repetitions of motor responses.

A previous study demonstrated an online learning effect of inhibition skill, including Go/Nogo and stop-signal tasks^26^. The protocol used required a greater amount of RI training repetition (including both the Go/Nogo and the stop signal tasks) than the current study, suggesting that greater trial repetition might unveil a significant training effect of RI skill. Another possible explanation is that we performed an unrecorded adaptive training of the Go and Go/Nogo tasks to confirm PC task operation proficiency, which may have obscured a definitive training effect. Although the non-significant within-training improvement of Go/Nogo RT could mask the meaning of the following across-session improvement in the current study, the across-session improvement of Go/Nogo RT may engage a specific feature of the offline consolidation process during RI skill learning. Because only the intersession intervals of the particular conditions improved the retest Go/Nogo RT regardless of sleep deprivation, it suggests that the improvements of Go/Nogo RT are not simply due to the continuous effect of online training. Instead, resistibility for online training and insidious delayed improvement could be a crucial RI skill property that might complicate the motor skill learning process.

Significant delayed improvements in RT after a 12- or 24-h intersession interval were observed in the groups that contained the elapsed daytime (8 a.m. to 8 p.m.) period regardless of whether those groups had a night’s sleep, a daytime nap, or SD. Moreover, the group-specific delayed improvements in Go/Nogo RT were independent of alertness changes due to SD prior to the retest session. Thus, we considered the elapsed time period in the 12-h time window from 8 a.m. to 8 p.m. to be the most likely source of the offline improvement in RT on the Go/Nogo RI task. In contrast to RI, no significant improvements across intersession interval were observed in HR or RT for the Go task in any of the groups, which is in agreement with previous studies^18^,^21^,^33^,^39^. Hence, Go task performance robustly reflects the essential motor acceleration component of motor skills and might suggest a ceiling effect of simple motor response skill improvement.

Go/Nogo RT showed significantly delayed improvements across a 12-h intersession interval during the daytime (groups C and D) but not the nighttime (groups A and B). These results highlight two potential characteristics of offline RI skill learning: (1) sleep/wake states are independent of offline RI skill improvement or (2) offline RI skill improvement occurs during a specific time window during the day (8 a.m. to 8 p.m.). However, the remaining problem is that the Go/Nogo RTs in all of the groups retested in the morning (groups A–B) did not improve, whereas they were faster in groups retested in the evening (groups C–F). This suggests that a potential time-of-day effect on Go/Nogo RT in the retest session makes it appear as though Go/Nogo RT improved offline. This is an unlikely scenario because significantly delayed improvements in Go/Nogo RT across the 24-h intersession interval in groups E and F suggest that there is a weak time-of-day effect on training–retest repetition or an influence of alertness on delayed Go/Nogo RT improvement. In addition, there was no detectable time-of-day effect on Go/Nogo RT in the initial training session. Besides, if the improvement in Go/Nogo RT is attributable to a simple diurnal variation in Go/Nogo RT in the retest session, the % improvement in groups A and B should show significant negative changes. As it is, Go/Nogo RT in groups A and B did not show significant intersession differences outside the margin of error. Furthermore, non-significant differences in % improvement of Go/Nogo RT among all of the groups suggested a low probability of an interaction between diurnal variation in the online retest (or training) performances and offline Go/Nogo RT improvement.

Although SD enhanced sleepiness at the retest session for groups B and F, there were no significant differences in delayed improvement rates in Go/Nogo RT compared to groups A and E, respectively. The non-significant intragroup differences in Go/Nogo RT among groups A–D clearly suggested sleep-independent delayed improvement in Go/Nogo RT. The non-significant intergroup differences in % improvement of Go/Nogo RT among groups C–F confirmed that there was no effect of alertness or intersession interval (12 or 24 h) on delayed Go/Nogo RT improvement (Fig. 6).

It should be noted that the difference in offline consolidation between the MST and PM skills could be causally related to the RI skill. The PM skill involves a greater RI component against the accelerating movement with respect to each randomly presented stimulus for a correct response. That is, the MST skill minimizes the inhibition effort by predicting the next movement due to the preplanned motor sequence. In addition, MST skill depends to a large extent on the explicit representation to motor outputs because subjects repeatedly push keys in a known sequence of “explicit awareness.” However, the PM skill was estimated by key press responses to a target that randomly appeared in one of a row of four locations^25^,^40^ and did not require explicit knowledge compared with the MST skill. Song *et al.* inferred from the offline learning properties of PM and MST skills that the sleep-dependent delayed consolidation of MST skill is acquired through its enriched explicit learning process^31^. Conversely, the implicit (unpredictable) learning process, which dominates PM skill, benefits from the daytime waking period^41^. Specific motor movements attributed to the primary motor cortex improved because of the offline skill learning process during waking, while motor responses dependent on explicit representation to motor outputs attributed to the hippocampus were improved by the offline skill learning process during sleep^31^. Furthermore, both explicit representation to motor outputs and implicit contextual information could support predictions regarding the forthcoming motor outputs and facilitate offline motor skill learning during sleep^42^. In line with this suggestion, RI skill improvement might occur via a sleep-independent offline learning process because the Go/Nogo task is not predictable and requires optimized participant responses.

RI involves inhibition of a prepotent motor response to a stimulus, which requires different neural systems from those involved in pure motor-sequence learning seen in MST skill that are known to show offline improvements during sleep. It has been assumed that RI and WM skills share a similar neural basis in the PFC^27^ because RI simultaneously requires high-order cognitive functions to optimize behaviors via top-down regulation from the PFC^43^,^44^,^45^. The left frontoparietal network comprises the IFG, pre-SMA, SMA, and DLPFC and is actively involved in RI processing^15^,^27^,^46^,^47^. RI and WM skills both seem to require higher executive PFC function, and WM skill improves offline to a greater degree during a sleep period compared to the same period of wakefulness^29^. However, RI may require different neuroplastic mechanisms. Kuriyama *et al.* (2008)^29^ utilized a *n*-back WM task to investigate a delayed improvement of WM skills and found sleep-dependent improvements. The *n*-back WM task requires subjects to acquire newly presented stimulus information and simultaneously requires the subject to explicitly maintain a stimulus sequence and respond to questions regarding what stimuli were presented *n* times before. The WM task dominantly activates the right DLPFC^48^,^49^, whereas the Go/Nogo task activates the left IFG and DLPFC^27^. In addition, WM skills seem to require a greater explicit cognitive component rather than an implicit cognitive component^50^. These differences between RI and WM might explain the timing difference in the offline skill consolidation process.

Many aspects of human physiology and behavior are under control of the circadian oscillation, although not to the extent seen in wild animals. Human cognition is fundamentally modulated by the circadian processes, and memory formation is deeply influenced by sleep-wake homeostasis^51^. The core molecular circadian oscillator exists as an interlocked transcriptional auto-regulatory feedback loop, including the *period* (*PER*) and *clock* (*CLK*) genes. Their expression patterns assist rhythmic activation of cellular signaling cascades such as the cyclic adenosine monophosphate (AMP)-mitogen-activated protein kinase (MAPK)-cAMP-responsive element-binding protein (CREB) pathway, which regulates both long-term memory formation and circadian rhythm per se^52^,^53^. This pathway is activated during nighttime sleep and is required during the consolidation and reconsolidation of hippocampus-dependent memory (e.g., declarative or explicit memory). Because RI is a nondeclarative and implicit skill, RI skill consolidation may be independent of nighttime cAMP–MAPK–CREB pathway activation.

The study has several potential limitations that made it difficult to clarify the skill consolidation properties of RI. First, our study did not allow disentanglement of the contributions of time-of-day effect and homeostatic effect of sleep deprivation on immediate and delayed improvements. Although a crossover experimental design would verify the interaction between time-of-day and training–retest repetition effects on Go/Nogo task performances, we utilized a between-group design to minimize the cumulative effect of repetitive learning of the RI skill. In addition, circadian effects on retesting are a major concern that was not entirely eliminated by the experimental setting. A possible solution to determine if the observed improvement in groups C–F was due to an offline consolidation effect over daytime or a circadian effect on retesting could be to include an additional control group in which participants are trained in the morning and tested 24 hours later. In this way, the consolidation interval would include a full daytime, and retesting would take place in the morning. Second, a consistent but not strictly controlled experimental setting could obscure the possible time-of-day (circadian) and/or alertness effect on Go and Go/Nogo task performances. Previous studies^54^,^55^ clearly suggested a circadian variation with higher cognitive task performances and alertness levels in the evening compared to the morning under a constant routine regimen. Some confounders associated with daily life environments could have diminished such sensitive time-of-day influences on task performances in the current study. Third, there was a difference in task difficulty between the Go and Go/Nogo tasks. Lower difficulty in the Go task resulted in a ceiling effect on task performance that could obscure the delayed (offline) learning of the simple response skill in the Go task. In addition, smaller trial repetitions of the Go and Go/Nogo tasks may also obscure immediate (online) training effects^25^,^26^, possible time-of-day effects^54^,^55^, and possible influences of alertness. However, the consistently higher HR supports the RT findings because of the trade-off between HR and RT. Finally, sleep during the inter-session interval was only evaluated by actigraphic recording. Although actigraphy is a simple and precise method for evaluating sleep duration^56^, polysomnography is a more accurate and reliable measurement to assess sleep duration and quality. Further research needs to be performed to determine the rigorous circadian effects on offline consolidation of RI skills and the longitudinal effect of RI skill training on human behavior and psychosocial function.

## Methods

### Participants

Seventy-nine healthy right-handed volunteers (46 females; mean age, 21.4 years; range, 20–26 years) with no previous history of neurological, psychiatric, sleep, or circadian rhythm disorders participated in the study. They were instructed to avoid psychoactive substances such as nicotine, alcohol, and caffeine for 24 h before the study period and throughout the experiment. They were asked to maintain a constant sleep-wake schedule from 1 week prior to the end of the experiment, and their adherence was confirmed by a sleep assessment questionnaire (Table 1). All participants provided written informed consent prior to their participation. The study protocol was designed in accordance with the Declaration of Helsinki and was approved by the Intramural Research Board of the National Center of Neurology and Psychiatry.

### Experimental design

The experiment consisted of a training session and a retest session with a 12- or 24-h intersession interval. Participants were randomly assigned to one of six experimental groups with different intersession interval conditions (groups A–F, Fig. 1). Participants performed an auditory Go/Nogo task in each training and retest session. All test sessions that took place after a night’s sleep or a daytime nap began at least 1 h after awakening to avoid a possible effect of sleep inertia on task performances^57^,^58^. Just before the initial training session, each subject performed a brief version of the auditory Go/Nogo task to become familiar with the PC procedure. Throughout the experimental period, all participants stayed in the laboratory and were monitored with an ambulatory wrist activity recorder (Actiwatch-L, Mini-Mitter Co., Inc. Bend, OR) designed to sense movement to distinguish between waking and sleeping states.

To determine whether a subsequent night’s sleep led to any marked improvement in Go and Go/Nogo performance, participants in groups A (*n* = 14 [8 females; mean age, 21.3 years; range, 20–24 years]) and B (*n* = 12 [6 females; mean age, 21.2 years; range, 20–25 years]) were trained at 8 p.m. and retested at 8 a.m. the next morning after a 12-h intersession interval. During this interval, group A participants slept in an isolated cabin under dimly lit conditions (<1 lux) from 11 p.m. to 7 a.m., and their sleep was monitored via infrared remote video and an ambulatory wrist activity recorder. Group B participants were deprived of nocturnal sleep and kept awake during the intersession interval in an isolated cabin under normal light conditions (200 lux).

To determine whether a subsequent daytime nap (3 h) led to any marked improvement in the Go and Go/Nogo performances, participants in groups C (*n* = 13 [6 females; mean age, 21.1 years; range, 20–24 years]) and D (*n* = 13 [6 females; mean age, 21.2 years; range, 20–24 years]), were trained at 8 a.m. and retested at 8 p.m. after a 12-h intersession interval. Group C participants were awake during the entire intersession interval, while group D participants were forced to nap in an isolated cabin under dimly lit conditions (<1 lux) from 1 p.m. to 4 p.m. During the nap period, they were monitored by infrared remote video and an ambulatory wrist activity recorder.

To confirm whether deprivation of the previous night’s sleep impaired Go and Go/Nogo performance improvements during the subsequent day, participants in group E (*n* = 13 [6 females; mean age, 21.2 years; range, 20–25 years]) were trained at 8 p.m., subjected to an immediate 8-h sleep period and wakefulness, and then retested at 8 p.m. the next day. During the night, group E participants slept in an isolated cabin under dimly lit conditions (<1 lux) and monitored in the same manner as those in groups A and D. Participants in group F (*n* = 14 [6 females; mean age, 21.2 years; range, 20–25 years]) engaged in the same experimental schedule as group E, but were deprived of nocturnal sleep and kept awake for the 24-h intersession interval.

An auditory Go/Nogo task was employed to elucidate the learning properties of RI skill^51^,^59^. An auditory Go task was also used to determine the simple learning properties of the motor response skill as a control. Three trial blocks of the Go/Nogo task (RI task) with three trial blocks of the Go task (simple response task) were run in each training and retest session in a randomized order to minimize the potential bias in task performances caused by inter-block interferences. Each trial block was separated with a 30-s inter-block interval. Each trial block consisted of 20 trials with inter-trial intervals (ITIs: average, 750 ms; range, 500–1000 ms). Participants listened to a sequence of 20 individual 50-ms tone stimuli at 70 decibels in a trial block via headphones. The sequence consisted of 15 low-pitched tone stimuli (75%) with 1000-Hz frequency and 5 high-pitched tone stimuli (25%) with 1200-Hz frequency. Participants had to respond to all tones by pressing a button as quickly and accurately as possible in the Go trial blocks. Participants had to respond to low-pitched tones by pressing a button as quickly and accurately as possible but were asked to withhold a response to the high-pitched tones in the Go/Nogo trial blocks. Thus, 20 and 15 responses were obtained in a trial block of the Go and Go/Nogo tasks, respectively. RT and HR were calculated in each block and session. Responses made after 500 ms from the offset of tone stimuli were considered faults.

At the beginning of each session, the subjective sleepiness of each participant was measured with a visual analog scale (VAS). The VAS consisted of a 100-mm-long horizontal line labeled “extremely alert” on the left and “extremely sleepy” on the right. Participants drew a vertical mark on the line at a point corresponding to their level of sleepiness. Higher scores indicated greater subjective sleepiness.

### Statistics

ANOVAs and multiple comparisons were used to examine task performance (RT and HR) and sleepiness level. Two-way, between-group (six groups)×within-trial block (three trial blocks), ANOVAs were conducted for Go and Go/Nogo performances during the online training session. Two-way, between-group (six groups)×within-session (two sessions), ANOVAs were conducted for Go and Go/Nogo performances and subjective sleepiness levels across the training–retest sessions. A one-way between-group ANOVA was conducted to examine group differences in offline improvement rates (% improvements) in Go and Go/Nogo performances. Pearson’s correlation coefficients were calculated to examine the influence of alertness (subjective sleepiness) on Go and Go/Nogo performances. Unpaired *t*-tests were used to examine the influence of time of day (8 a.m. or 8 p.m.) on task execution on online Go and Go/Nogo performances. Stepwise multiple linear regression analyses were performed to identify independent predictors of offline improvements in Go/Nogo performances, such as sleep state (sleep or wakefulness), time of day (nighttime, daytime, or whole-day), and training–retest interval (24-h or 12-h) using a dummy variable adjustment method. A variance inflation factor (VIF)≥10 was regarded as significantly serious multi-collinearity. All tests were two-tailed. The results are shown as the mean and standard error of the mean (SEM). A *p*-value of 0.05 was considered statistically significant, but a corresponding Bonferroni-adjusted *p* value for each statistical level was set for each *post-hoc* test. SPSS 16.0 J for Windows (SPSS, Inc., Chicago, IL) was used for the statistical analyses.

## Additional Information

**How to cite this article**: Honma, M. *et al.* Sleep-independent offline consolidation of response inhibition during the daytime post-training period. *Sci. Rep.* **5**, 10362; doi: 10.1038/srep10362 (2015).

## Figures and Tables

**Table 1:** Sleep variables.

**Group**	**Habitual sleep onset time**	**Habitual awakening time**	**Habitual sleep duration (h)**
A	0:13 (13 min)	7:26 (14 min)	7.36 (0.21)
B	0:05 (15 min)	7:15 (11 min)	7.25 (0.20)
C	0:23 (14 min)	7:29 (16 min)	7.19 (0.28)
D	0:06 (23 min)	7:14 (23 min)	7.21 (0.41)
E	0:23 (13 min)	7:25 (15 min)	7.09 (0.27)
F	0:09 (14 min)	7:08 (17 min)	7.01 (0.41)

The SEMs are shown in parentheses.

**Table 2:** HRs and RTs for the Go task.

	**HR (%)**		**RT (ms)**	
**Group**	**Training**	**Retest**	**Training**	**Retest**
A	98.71 (0.41)	99.05 (0.49)	299.7 (14.1)	304.4 (14.0)
B	99.67 (0.22)	99.33 (0.38)	315.0 (15.2)	320.5 (15.1)
C	98.85 (0.35)	98.72 (0.71)	316.8 (14.6)	315.4 (14.5)
D	98.51 (0.38)	98.80 (0.72)	296.3 (14.6)	292.9 (14.5)
E	99.29 (0.29)	99.76 (0.15)	316.9 (15.1)	326.1 (15.0)
F	99.81 (0.11)	99.74 (0.16)	299.1 (14.6)	310.1 (14.5)

HR, hit rate; RT, response time. The SEMs are shown in parentheses.
